# MicroRNA-195 Activates Hepatic Stellate Cells In Vitro by Targeting Smad7

**DOI:** 10.1155/2017/1945631

**Published:** 2017-08-27

**Authors:** Li-Ying Song, Yu-Tao Ma, Cui-Fang Wu, Chun-Jiang Wang, Wei-Jin Fang, Shi-Kun Liu

**Affiliations:** ^1^Department of Pharmacy, The Third Xiangya Hospital, Central South University, Changsha, Hunan, China; ^2^Department of Pharmacy, Shaoxing Seventh People's Hospital, Shaoxing, Zhejiang, China

## Abstract

**Background and Aim:**

Aberrant activation of the TGF-*β*1/Smad pathway contributes to the activation of hepatic stellate cells (HSCs). MicroRNA-195 has been shown to regulate the activation of HSCs. The aim of this study was to investigate the role of miRNA-195 in HSCs activation.

**Methods:**

A liver fibrotic rat model induced by diethylnitrosamine was established. Dual luciferase reporter assays were performed to verify that Smad7 was the target of miRNA-195. The expression levels of miR-195, Smad7, and *α*-SMA in HSC-T6 transfected, respectively, with miR-195 mimic, inhibitor, or control were measured by qRT-PCR. The protein expression of Smad7 was detected by Western blot analysis.

**Results:**

Enhanced miR-195 and decreased Smad7 were observed in diethylnitrosamine-induced liver fibrotic rats (*P* < 0.05). Dual luciferase reporter assays showed that the miR-195 mimic significantly suppressed the luciferase activity of a reporter plasmid carrying the binding site of miR-195 on the 3′UTR of Smad7 (*P* < 0.05). The miR-195 mimics activated HSCs, further elevated miR-195 and *α*-SMA (*P* < 0.01), and reduced the Smad7 level (*P* < 0.05). The miR-195 inhibitors blocked the activation of HSCs, reduced the expression of miR-195 and *α*-SMA (*P* < 0.01), and upregulated the expression of Smad7 (*P* < 0.05).

**Conclusion:**

Collectively, we demonstrated that miRNA-195 activated HSCs by targeting Smad7.

## 1. Introduction

The prevalence of liver fibrosis has increased due to hepatitis B virus, alcohol toxicity, inflammation, and diabetes via increased synthesis and decreased degradation of extracellular cell matrix (ECM) in hepatocytes [1–3]. This condition is associated with an increased risk of diseases, such as hepatocellular carcinoma (HCC), which is a leading cause of death worldwide [4]. Hepatic stellate cell (HSC) activation plays a vital role in the formation of ECM, and these cells are activated to myofibroblast- (MFB-) like cells progressively during liver fibrosis [5, 6]. *α*-Smooth muscle actin (*α*-SMA) is a key marker of HSC activation. Transforming growth factor-*β*_1_ (TGF-*β*_1_), a major profibrogenic mediator, activates HSCs through the Smad2/3 pathway [7]. However, the mechanisms underlying HSC activation-induced liver fibrosis, as well as structural and functional abnormalities, remain incompletely understood.

MicroRNAs (miRNAs), endogenous noncoding RNA molecules of 20–22 nucleotides in length, act as negative regulators of gene expression by inhibiting protein translation or inducing mRNA degradation [8]. Growing evidence has demonstrated that mammalian miRNAs are involved in various biological processes, such as differentiation, proliferation, immune responses, oxidative stress resistance, and carcinogenesis [9]. Moreover, most of the studies have shown that miRNAs regulate the activation of HSCs in liver fibrosis. For example, microRNA-378 limited the activation of HSCs and liver fibrosis by regulating expression of its target glioma-associated oncogene family zinc-finger 3 gene [10]. Overexpression of miR-17-5p promoted HSCs proliferation and activation by activating the Wnt/*β*-catenin pathway [11]. MiR-15b and miR-16 were downregulated when HSCs were activated, and their overexpression induced HSCs apoptosis and cell cycle arrest through caspase and Bcl-2 signaling pathways [12]. Furthermore, miR-194 and miR-150 regulated ECM synthesis and HSCs activation by reducing Ras-related C3 botulinum toxin substrate 1 and c-myb gene [13]. Other studies showed that miRNA-195 was upregulated in hexahydro-1,3,5-trinitro-1,3,5-triazine- (RDX-) treated rats with liver fibrosis [14].

MiR-195, an important member of the miR-15 family, plays a crucial role in regulating cell cycle progression and cell apoptosis [15]. MiR-195 plays various roles in different diseases. In primary peritoneal carcinoma and tongue squamous cell carcinoma, miR-195 was decreased. In adrenocortical carcinoma, breast cancer, and chronic lymphoblastic leukemia, however, miR-195 was increased [16, 17]. In HCC, miR-195 inhibited the cell cycle by downregulating cell cycle protein D, the transcription factor E2F3, and cyclin-dependent kinase 6 [18–20]. With regard to HSCs, Sekiya found that, during primary HSCs (pHSCs) activation, miR-195 was downregulated on the 10th day compared with the 1st day, and miR-195 could decrease cyclin E expression and inhibit cell proliferation induced by interferon-B [21]. Duan and Chen demonstrated that miR-195 was upregulated by TGF-*β*1 in a dose-dependent manner enhancing glioma cell invasion and proliferation in U87 cells [22]. Growing evidence has demonstrated that miR-195 plays an important role in the neoplastic disease; however, studies on the relationship between miR-195 and liver fibrosis are far from enough.

TGF-*β* is a central regulator of HSC activation, and the TGF-*β*_1_/Smad pathway plays a pivotal role in promoting liver fibrosis [23]. The TGF-*β* pathway involves the receptor-activated Smads (Smad2 and Smad3) through direct serine phosphorylation of the COOH termini by T*β*RI upon TGF-*β* binding [24]. During acute liver injury or external stimuli, TGF-*β*_1_ promotes collagen synthesis in the activated HSCs via the pSmad2L/C and pSmad3C pathways. Smad7, an inhibitory factor, is induced by pSmad3C and terminates the fibrogenic phospho-Smad signaling and negatively regulates fibrogenic signaling to avoid excess ECM deposition. In contrast, Smad7 cannot be induced by the pSmad3L pathway in chronic liver injury, leading to constitutive fibrogenesis in MFBs [25–27]. The precise mechanism underlying the low expression of Smad7 in HSC activation is incompletely understood.

MicroRNA target prediction analyses indicated that miR-195 could target the 3′UTR region of Smad7. Previous studies showed that miR-195 could bind to Smad7 and its target site was located within 1611 and 1630 nt of Smad7 mRNA 3′-UTR and miR-195 was possibly a tumor promoter in U87 cells by targeting Smad7 [22, 28]. However, the precise roles of miR-195 in the TGF-*β*_1_/Smad pathway in HSCs remain unclear to date. In this report, we confirmed that miR-195 was increased in TGF-*β*_1_-induced HSCs as well as in diethylnitrosamine- (DEN-) induced rat liver fibrotic tissues. Overexpression of miR-195 in HSC-T6 suppressed Smad7 expression and upregulated *α*-SMA expression induced by TGF-*β*_1_, and inhibition of miR-195 suppressed *α*-SMA expression and increased Smad7 expression induced by TGF-*β*_1_. Furthermore, miR-195 activated HSCs through direct targeting of Smad7. Identification of miR-195 as a novel marker involved in HSC activation and liver fibrosis supports its potential utility as a therapeutic target.

## 2. Materials and Methods

### 2.1. Materials

DEN, TGF-*β*_1_, and MTT were obtained from Sigma (St. Louis, MO, USA). All miR-195 mimic, mimic controls, inhibitor, inhibitor control, and primers for miRNA PCR were purchased from RiboBio (Guangzhou, China). The transfection reagent siRNA-Mate was purchased from GenePharma (Shanghai, China). Oligonucleotide primers for *α*-SMA, GAPDH, U6, and Smad7 were designed by Sangon Biotech (Shanghai, China). Primary antibodies (rabbit anti-Smad7 and mouse anti-GAPDH) were obtained from Abcam (Cambridge, MA, USA). The secondary goat anti-rabbit and goat anti-mouse IgG antibody were obtained from (Calbiochem, CA, USA).

### 2.2. DEN-Induced Rat Model of Liver Injury

The rat model of liver injury was established as previously described [29]. Male Sprague-Dawley rats (180–220 g) were provided by the Experimental Animal Center of The Third Xiangya Hospital, Central South University. After a 5-day acclimatization period, 12 rats were randomly divided into two groups: control group (given the same volume saline as the DEN model group) and the DEN group (given 0.2% DEN (10 *μ*g/kg) by oral gavage five times a week until the 4th week). DEN was diluted with saline. Six rats from each group were sacrificed at the end of week 4. Blood was collected for laboratory analysis of serum alanine aminotransferases (ALT) and aspartate aminotransferases (AST). Liver tissues were harvested for hematoxylin and eosin (H&E) and Masson staining. All animal experiments were performed according to the Guidelines of Animal Experiments from the Committee of Medical Ethics at the National Health Department of China and were approved by Central South University.

### 2.3. Histopathological Examination

Histopathological examination was performed as described previously to observe the pathological changes in the liver tissues and determine the extent of liver fibrosis [30–32]. Samples of liver tissue were fixed in 10% buffered formalin, routinely embedded in paraffin, cut into 4 mm thick sections, and stained with hematoxylin and eosin (HE) and Masson trichrome staining for examination by light microscopy. A blinded investigator from pathology department evaluated the slides for inflammation existence and fibrosis.

### 2.4. Cell Culture

The rat HSC-T6 cell line was donated by the Department of Pathophysiology of Xiangya Medical College. Cells were maintained in DMEM containing 10% fetal bovine serum, 100 U/ml penicillin G sodium salt, and 100 U/ml streptomycin sulfate (Gibco, CA, USA) and incubated at 37°C under an atmosphere of 5% CO_2_. Exponentially growing cells were treated with 1, 2, 4, 6, and 8 ng/mL TGF-*β*_1_ for 24 h, 48 h, and 72 h. Then, MTT assays were performed as described previously to evaluate cell proliferation. The exponentially growing cells were treated with 2, 5, and 10 ng/ml TGF-*β*_1_ (Sigma, USA) for 24 h to select the optimal concentration for study. Cells were harvested for RNA/miRNA isolation, and whole cell extracts were subjected to western blot analysis.

### 2.5. miRNA Transfection

Cells were seeded in a 6-well plate at a density of 1 × 10^6^ cells per well. The following day, the medium was replaced with Opti-MEM (Invitrogen, USA), and the cells were transfected with an miR-195 inhibitor (200 nM) and miRNA inhibitor control (200 nM) (RiboBio, China) using the transfection reagent siRNA-Mate (GenePharma, China) for 24 h. After 24 h of transfection, the medium was replaced with DMEM containing 10% FBS, and TGF-*β*_1_ was added at a concentration of 5 ng/ml. We select a concentration of 5 ng/ml for TGF-*β*_1_, because TGF-*β*_1_ at a concentration of 5 ng/ml and 10 ng/ml could both significantly increase miR-195 expression and there were no differences between them. Cells were additionally transfected with miR-195 mimics (100 nM) and miRNA mimic controls (200 nM) using siRNA-Mate for 24 h.

### 2.6. Quantitative Real-Time PCR

Total RNA was extracted from HSC-T6 cells with TRIzol (OMEGA, CA, USA) and a miRNeasy Mini kit (Toyobo, Osaka, Japan). cDNA was synthesized using a Rever Tra Ace qPCR RT Kit (Toyobo, Osaka, Japan) in accordance with the manufacturer's instructions. Gene expression was measured with real-time PCR using cDNA and a SYBR Green Real-Time PCR Master Mix (Toyobo, Osaka, Japan). The RT reaction for miR-195 was performed using a TaqMan MicroRNA Assay (Toyobo, Osaka, Japan) according to the manufacturer's instructions. GAPDH and U6 snRNA (Toyobo, Osaka, Japan) levels were measured and used to normalize the relative abundance of mRNA and miRNA, respectively. The relative expression level (2^−ΔΔCt^) of target mRNA and miRNA was calculated in accordance with previous reports.

### 2.7. Protein Extraction and Western Blot

Tissues were lysed with ice-cold RIPA lysis buffer containing 1% PMSF after centrifugation as described previously. Protein concentrations in the samples were measured with a spectrophotometer. Antibodies used in this study included GAPDH and Smad7 (Abcam, Cambridge, MA, USA). The total protein (20 *μ*g) was separated by 10% SDS polyacrylamide gel electrophoresis. The resolved proteins were then transferred to a polyvinylidene fluoride (PVDF) membrane, which was subsequently blocked for 3 h at room temperature with 5% skim milk in TBS-T (10 mM Tris, 150 mM NaCl, and 0.1% Tween-20). After washing with TBS-T, the membrane was incubated with the following antibodies overnight at 4°C: anti-Smad7 at a 1 : 1000 dilution and anti-GAPDH at a 1 : 10000 dilution. The secondary antibodies (Jackson, USA) (goat anti-rabbit and goat anti-mouse IgG horseradish peroxidase conjugated antibody) were used at a 1 : 4000 dilution to detect the target protein. The reaction was visualized on X-ray medical film after incubation of the membranes with luminol-based chemiluminescence reagent (Invitrogen, USA) for 5 min. Experiments were carried out in triplicate at least three times.

### 2.8. Cell Proliferation Assay

Cells were seeded in a 96-well plate at a density of 1 × 10^3^ cells per well and treated with 1, 2, 4, 6, and 8 ng/mL TGF-*β*1 for 24 h, 48 h, and 72 h. HSC-T6 cells were transfected with miR-195 mimics, miR-195 mimic controls, miR-195 inhibitors, and miR-195 inhibitor controls as described above. MTT assays were performed as described previously to evaluate cell proliferation using the protocol from the MTT cell proliferation assay kit (Beyotime Institute of Biotechnology). Optical density (OD) was measured at 490 nm on an ELx800NB microplate reader (Bio-Rad, USA). All experiments were performed in triplicate and repeated at least three times.

### 2.9. Luciferase Activity Assay

One day before transfection, HEK 293T cells were seeded in 24-well culture plates in culture medium without antibiotics. The 3′UTR region of the Smad7 gene was cloned into the pMIR-REPORT™ Luciferase plasmid (Applied Biosystems) to generate the pMIR-Smad7 and pMIR-Smad7-Mut vectors. Using Lipofectamine 2000 (Invitrogen), HEK 293T cells were transfected with a mixture of the pMIR-Smad7 vector and either a miR-195 mimic (50 nM) or the same concentration of scrambled miRNA (miRNA mimic negative control) as a negative control. At 48 h after transfection, the cells were harvested and tested with a Dual Luciferase Reporter Assay System (Promega) in accordance with the manufacturer's protocol. The pMIR-REPORT*β*-gal control plasmid was used for transfection normalization. All firefly luciferase activity data are presented as the mean ± SD from at least three experiments.

### 2.10. Statistical Analysis

Results are expressed as the mean ± SD. Statistical significance between the control and treated groups or subgroups was analyzed by Newman-Student-Keuls or one-way ANOVA. The differences between subgroups were analyzed by two-sample Student's *t*-tests. Data was considered as significant when *P* values are <0.05. Statistical analyses were performed using IBM SPSS Statistics software 21.0.

## 3. Results

### 3.1. MiR-195 Increased in DEN-Induced Liver Injury

**Figure 1:**
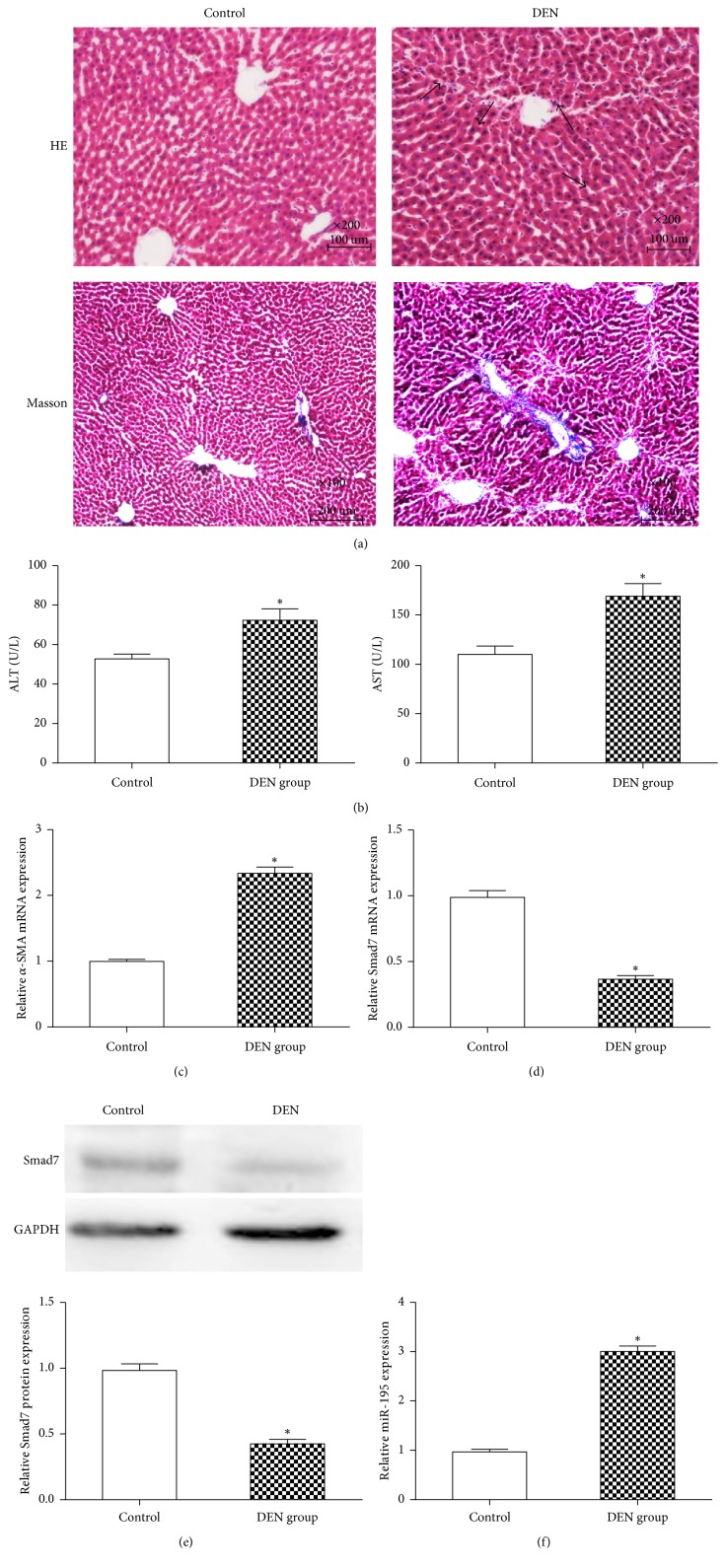
*MiR-195 and Smad7 expression in DEN-induced hepatic fibrotic tissues and control tissues*. (a) Hepatic fibrotic and control tissues were detected by hematoxylin and eosin (H&E) staining (×200) and Masson staining (×100) to assess liver fibrosis. Black arrows indicate hepatic steatosis, necrosis, and inflammatory cell infiltration. (b)  The plasma  concentrations of ALT and AST in rats. (c) The mRNA levels of *α*-SMA in rat liver tissues were detected with qRT-PCR. (d) The mRNA levels of Smad7 in rat liver tissues were detected with qRT-PCR. (e) The protein levels of Smad7 in rat liver tissues were detected with Western blotting. (f) The miR-195 expression in hepatic fibrotic tissues was detected with qRT-PCR. Each value represents the mean ± SD of three experiments. ^*∗*^*P* < 0.05 versus control.

DEN is a potent toxic compound that can cause necrosis and subsequent fibrosis in the liver. To generate the experimental model of hepatic fibrosis, we exposed SD rats to DEN five times per week for 4 weeks. H&E staining and Masson staining showed increased centrilobular necrosis and apoptotic hepatocytes in DEN livers compared with the control group (Figure 1(a)). The plasma concentrations of ALT and AST in the DEN group significantly increased compared to those rats in the control group (Figure 1(b)). The mRNA level of *α*-SMA was higher in livers of the DEN group than those in the control group (Figure 1(c)). These results indicated that DEN induced hepatic fibrosis in rats.

The mRNA and protein expressions of Smad7 were both significantly decreased in liver tissues of the DEN group compared with the control group (Figures 1(d) and 1(e)). MiR-195 was significantly increased in the liver tissues of the DEN group compared with the control group (Figure 1(f)).

### 3.2. TGF-*β*_1_ Induced *α*-SMA, miR-195, and Smad7 Transcription in HSCs

**Figure 2:**
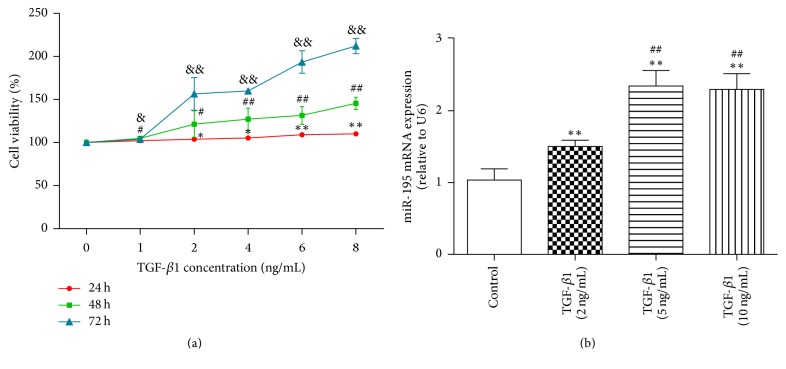
*TGF-β*_*1*_ *promotes HSC-T6 proliferation and miR-195 transcription*. (a) MTT assays for cell proliferation with different concentrations of TGF-*β*_1_ (1 ng/mL, 2 ng/mL, 4 ng/mL, 6 ng/mL, and 8 ng/mL) at 24 h, 48 h, and 72 h. ^*∗*^*P* < 0.05 versus control (24 h); ^#^*P* < 0.05, ^##^*P* < 0.01 versus control (48 h); ^&^*P* < 0.05, ^&&^*P* < 0.01 versus control (72 h). (b) miR-195 expression in HSC-T6 cells treated with TGF-*β*_1_ (2 ng/mL, 5 ng/mL, and 10 ng/mL) for 24 h. ^*∗∗*^*P* < 0.01 versus control. ^##^*P* < 0.01 versus TGF-*β*_1_ (2 ng/mL). Each value represents the mean ± SD of three experiments.

**Figure 3:**
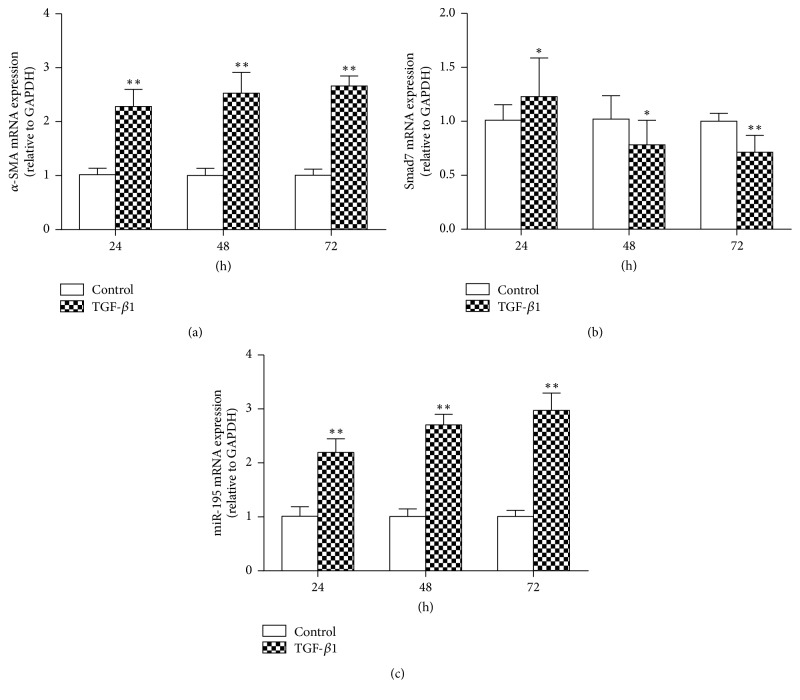
*TGF-β*_*1*_ *promoted α-SMA, miR-195, and Smad7 transcription in HSCs treated with 5 ng/mL TGF-β*_*1*_ *for 24 h, 48 h, and 72 h*. (a) qRT-PCR analysis of *α*-SMA expression in HSC-T6. (b) qRT-PCR analysis of Smad7 expression in HSC-T6. (c) qRT-PCR analysis of miR-195 expression in HSC-T6. Each value represents the mean ± SD of three experiments. ^*∗*^*P* < 0.05, ^*∗∗*^*P* < 0.01 versus control.

To determine the optimal TGF-*β*_1_ concentrations for HSC-T6 proliferation and miR-195 expression, HSC-T6 was treated with different concentrations of TGF-*β*_1_ (1 ng/mL, 2 ng/mL, 4 ng/mL, 6 ng/mL, and 8 ng/mL) at 24 h, 48 h, and 72 h. Then, cell proliferation was measured by MTT assays. MTT analyses showed that TGF-*β*_1_ promoted HSC proliferation in a time-dependent and concentration-dependent manner (Figure 2(a)). Then, HSC-T6 was treated with different concentrations of TGF-*β*_1_ (2 ng/mL, 5 ng/mL, and 10 ng/mL) for 24 h to detect the miRNA-195 expression.  Figure 2(b) shows that TGF-*β*_1_ increased miR-195 expression at 5 ng/mL and 10 ng/mL, TGF-*β*_1_ significantly increased miR-195 expression, and there were no differences between them. Therefore, we selected 5 ng/mL as the experimental concentration. HSC-T6 was treated with 5 ng/mL TGF-*β*_1_ for 24 h, 48 h, and 72 h, and qRT-PCR showed that TGF-*β*_1_ significantly increased *α*-SMA and miR-195 expression compared to the control group at 24 h, 48 h, and 72 h (*P* < 0.01) (Figures 3(a) and 3(c)). Compared with the control group, TGF-*β*_1_ increased Smad7 mRNA expression at 24 h (*P* < 0.05); however, TGF-*β*_1_ reduced Smad7 mRNA expression at 48 h and 72 h (*P* < 0.05, *P* < 0.01) (Figure 3(b)), indicating an initial increase and later decline. These data clearly indicated that elevated levels of miR-195 played a vital role in TGF-*β*_1_-activated HSCs.

### 3.3. MiR-195 Promotes HSC Proliferation and Modulates TGF-*β*_1_-Mediated *α*-SMA and Smad7 Expression

**Figure 4:**
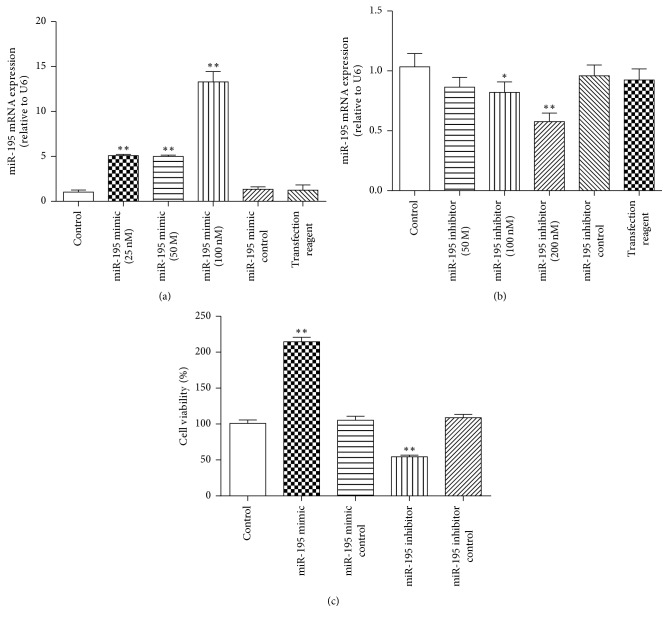
*Transfection effects of different concentration of miR-195 mimic and inhibitor in HSC-T6 and the cell viability transfected with miR-195 mimic and inhibitor*. (a) miR-195 expression was determined in HSC-T6 transfected with different concentrations of miR-195 mimic for 24 h by qRT-PCR. (b) miR-195 expression was determined in HSC-T6 transfected with different concentrations of miR-195 inhibitor for 24 h by qRT-PCR. (c) MTT assays for cell viability in HSC-T6 transfected with miR-195 mimic (100 nM)/inhibitor (200 nM). Each value represents the mean ± SD of three experiments. ^*∗*^*P* < 0.05, ^*∗∗*^*P* < 0.01 versus control.

**Figure 5:**
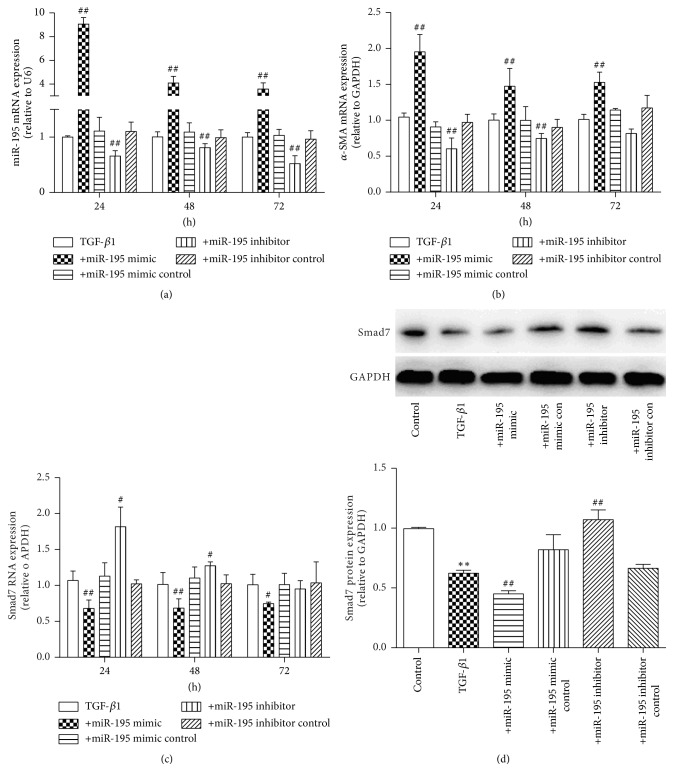
*Effects of miR-195 on α-SMA and Smad7 in HSC-T6 treated with TGF-β*_*1*_ *(5 ng/ml) for 24 h, 48 h, and 72 h*. (a) miR-195 expression was determined in HSC-T6 transfected with miR-195 mimic (100 nM) or miR-195 inhibitor (200 nM) by qRT-PCR. (b) *α*-SMA mRNA expression was determined in HSC-T6 transfected with miR-195 mimic (100 nM) or miR-195 inhibitor (200 nM) by qRT-PCR. (c) Smad7 mRNA expression was determined in HSC-T6 transfected with miR-195 mimic (100 nM)/inhibitor (200 nM) by qRT-PCR. (d) Smad7 protein expression was determined in HSC-T6 transfected with miR-195 mimic (100 nM)/inhibitor (200 nM) by Western blot. Each value represents the mean ± SD of three experiments. ^*∗*^*P* < 0.05, ^*∗∗*^*P* < 0.01 versus control; ^##^*P* < 0.01 versus TGF-*β*_1_.

To further establish the potential role of miR-195 in HSC activation, HSC-T6 was treated with miR-195 mimics (25 nM, 50 nM, and 100 nM), miR-195 mimic controls, miR-195 inhibitors (50 nM, 100 nM, and 200 nM), and miR-195 inhibitor controls for 24 h, and qRT-PCR was used to measure miR-195 expression. The data showed that 100 nM of the miR-195 mimic significantly increased the miR-195 level in HSC-T6, and 200 nM of the miR-195 inhibitor significantly reduced the miR-195 level in HSC-T6 (Figures 4(a) and 4(b)). HSC-T6 was treated with TGF-*β*_1_ and miR-195 mimic or miR-195 inhibitor, and we examined their effects on miR-195, *α*-SMA, and Smad7 expression. Enhanced miR-195 induced by TGF-*β*_1_ was significantly decreased in the presence of the miR-195 inhibitor, and the miR-195 mimic could increase the miR-195 level compared with the TGF-*β*_1_ group at 24 h, 48 h, and 72 h (Figure 5(a)). Enhanced *α*-SMA mRNA induced by TGF-*β*_1_ was significantly decreased in the presence of the miR-195 inhibitor at 24 h and 48 h and the miR-195 mimic could increase the *α*-SMA mRNA expression compared with the TGF-*β*_1_ group at 24 h, 48 h, and 72 h (Figure 5(b)). These results suggested that miR-195 induced *α*-SMA expression in activated HSCs. The miR-195 mimic could decrease Smad7 mRNA at 24 h, 48 h, and 72 h and decrease protein expression compared with the TGF-*β*_1_ group at 24 h; at the same time, the miR-195 inhibitor could increase Smad7 mRNA at 24 h and 48 h and increase Smad7 protein expression in comparison with the TGF-*β*_1_ group (Figures 5(c) and 5(d)). Our research showed that miR-195 inhibitor did not reduce the level of *α*-SMA or elevate the level of Smad7 at 72 h, we conclude that miR-195 inhibitor could not regulate the *α*-SMA and Smad7 expression with a time-dependent manner, on one aspect, the transfection reagents play the best effect at different time in different cells, and on the other aspect, TGF-*β*_1_ could significantly reduce the Smad7 level and increase *α*-SMA level at a time-dependent manner which may counteract the transfection reagents role in 72 h.

### 3.4. Smad7 Was the Target of miR-195

**Figure 6:**
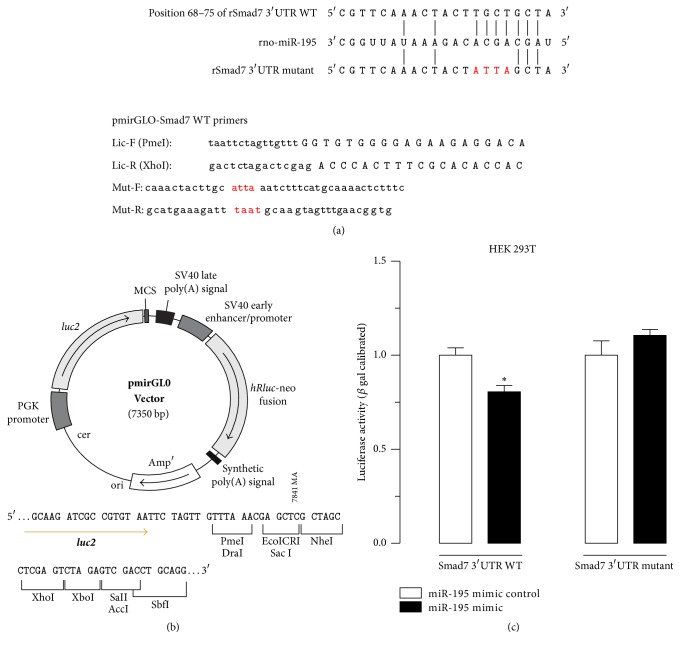
*Interaction of miR-195 with the 3*′*UTR of Smad7 inHEK 293T cells*. (a) miRNA binding sites and mutation site in the 3′UTR of Smad7 mRNA based on miRDB. Primer sequence for the PCR of Smad7 3′UTR MRE (WT) and the mutant (Mut). (b) Based on the pairing sites, corresponding luciferase reporter vectors were designed. (c) The graph depicts luciferase activity in cells transfected with Smad7 3′UTR WT or Smad7 3′UTR Mut as well as the miR-195 mimic or miR-195 mimic control. ^*∗*^*P* < 0.05 versus miR-195 mimic control.

To determine whether Smad7 was the target of miR-195, the 3′UTR of the Smad7 mRNA target region was cloned into the pMIR-REPORT luciferase plasmid, and the construct was cotransfected into HEK 293T cells, along with the miR-195 precursor or miR-NC (Figures 6(a) and 6(b)). Cotransfection of the *β*-gal reporter control plasmid was performed to monitor transfection efficiency. Notably, the miR-195 precursor significantly reduced the luciferase activity driven by wild-type 3′UTR of Smad7 compared with miR-NC in HSCs but did not affect luciferase activities of mutant type Smad7 3′UTR and the empty vector (Figure 6(c)). These findings collectively indicated that Smad7 was a direct target of miR-195.

## 4. Discussion

The aberrant activation of HSCs and subsequent continuous collagen fiber deposition is the central link to liver fibrosis and hepatic injury [33]. DEN is the most commonly used chemical to induce liver fibrosis in rats, and we used it to establish a liver fibrosis rat model [34]. HSC-T6 was derived from SD rat hepatic stellate cells transferred with antigen of SV40, so it possesses both the characteristics of quiescent HSC and the proliferation and activation ability of myofibroblasts. These cells could express *α*-SMA and extra cellular matrix; thus, we selected HSC-T6 as the liver fibrosis model in vitro. Liver fibrosis is a complicated pathological process. Various cytokines and growth factors, including transforming growth factor- (TGF-) *β*, platelet-derived growth factor B and D (PDGF-B and PDGF-D), endothelin-1, and tumor necrosis factor (TNF)-*α*, are involved in fibrogenesis. TGF-*β*_1_ was shown to be the fibrogenic master cytokine [35, 36]. TGF-*β*_1_ could activate HSCs to MFBs, accelerate matrix gene expression, decrease matrix degradation, and disrupt homeostasis, leading to collagen deposition. At the same time, activation of HSC can promote the secretion of TGF-*β*_1_, forming a positive feedback loop and accelerating liver fibrosis [37]. Therefore, we chose TGF-*β*_1_ to simulate liver fibrosis in vitro.

TGF-*β*_1_ predominantly promotes liver fibrosis by activating the downstream Smad signaling pathway, and TGF-*β*_1_/Smads have been considered to be a major player in liver fibrosis. Smad7, an inhibitory factor, is induced by TGF-*β*_1_ and negatively regulates fibrogenic signaling to prevent excess ECM deposition. Smad7 cannot be induced by the pSmad3L pathway in chronic liver injury, leading to constitutive fibrogenesis in MFBs; thus, the low expression of Smad7 in liver fibrosis may promote liver fibrosis progression. Previous studies have additionally shown that loss of Smad7 expression results from epigenetic modifications, including microRNAs, histone modification, promoter hypermethylation, and Smad phospho-isoforms related to Smad7 expression [5, 38–40]. MicroRNA target prediction analysis indicated that miR-195 can target the 3′UTR region of Smad7. Other studies showed that miR-195 could bind to Smad7 and regulate Smad7 expression in U87 cells and Caco-2 cells [22, 28]. Thus, our study aimed to determine whether miR-195 regulated HSC activation and Smad7 expression. Our study showed that miR-195 expression was upregulated in liver fibrotic tissues compared with the control tissues in vivo. The results indicated that TGF-*β*_1_ could stimulate HSCs activation and proliferation and promote miR-195 transcription. We found that TGF-*β*_1_ increased Smad7 mRNA expression at 24 h but decreased Smad7 protein expression at 24 h and then decreased Smad7 mRNA expression at 48 h and 72 h, which was consistent with other studies. Yoshida and Matsuzaki indicated that Smad7 was transiently increased in a rat liver injury model and then decreased with chronic liver injury [41]. Therefore, Smad7 was shown to be an immediate early gene that maintains the balance of TGF-*β*_1_/Smad signaling pathway by inhibiting signal transduction proteins, such as Smad2 and Smad3.

Furthermore, we assessed this hypothesis using dual luciferase reporter experiments. Oligonucleotides containing the human wild type and mutant Smad7 3′UTR target sequence were annealed and cloned into the pMIR-Report Luciferase plasmid. Our preliminary experiments showed that relative luciferase activity in transfected HSC-T6 was low; therefore, we utilized HEK 293T cells. The results confirmed that Smad7 was a target of miR-195, and miR-195 bound directly to the 3′UTR region of Smad7 mRNA. Previous studies showed that miRNAs could be utilized as a novel fibrosis biomarker. However, because it is difficult to obtain liver tissue samples, our future studies will investigate the specific role of serum/plasma miR-195 in regulating HSC activation and liver fibrosis.

In conclusion, our study provides new clues for the role of miR-195 in HSCs activation, and our results demonstrated that miR-195 activates HSCs by targeting Smad7 and promoting HSCs activation and proliferation. These findings indicate that miR-195 is a potential biomarker of hepatic pathogenesis and a possible therapeutic agent for treating liver diseases.
